# National Rural Health Mission: Time to Take Stock

**DOI:** 10.4103/0970-0218.55268

**Published:** 2009-07

**Authors:** Arun Kumar Sharma

**Affiliations:** Department of Community Medicine, University College of Medical Sciences, Dilshad Garden, Delhi - 110 095, India

The poor performance of the Indian Public Health System is widely acknowledged. Analysts have attributed this failure to a number of factors, which include almost all the components that make a system functional, that is, infrastructure, human resources, logistics, and participation of the community. However, some attribute this failure primarily to low and declining public investment in healthcare and secondarily to structural and managerial weaknesses in the system.

After groping with the challenges for decades, the planners have come up with a comprehensive mission-oriented approach to revamp the rural healthcare delivery system, which was aptly named National Rural Health Mission (NRHM) [Box 1]. The mission was launched on 12 April, 2005, to be completed in a time frame of seven years.(1) By the end of 2008, the mission has lived half of its life. It is the right time to take stock of the gains and achievements and critically analyze the failures and lapses.

Large variations exist in the health system and its performance, which is clearly evident in the fact that some states have been performing better than others in the pre-NRHM period. A need was felt by the Mission to bring the states that had been lagging behind at par. In order to provide an impetus to the primary healthcare infrastructure, so that the delivery of family welfare services could be made more efficient, the Empowered Action Group (EAG) was constituted by order of the Government of India on 20 March, 2001, as an administrative mechanism, for the purpose of closely monitoring the implementation of the family welfare program.(2) Initially it included eight states. In spite of a single window clearance mechanism for approving schemes to finalize strategies and address gaps in the ongoing programs, the states failed to show a desirable improvement in health indicators. Thus the mission focused on 18 poorly performing states, which included eight North Eastern states, eight Empowered Action Group (EAG) states, and the hilly states of Himachal Pradesh and Jammu and Kashmir.(3)

**Box 1:** National Rural Health Mission

**Aims**
The National Rural Health Mission aims at providing accessible, affordable, effective, accountable, and reliable healthcare to all citizens and in particular to the poorer and vulnerable sections of the population; consistent with the outcomes envisioned in the Millennium Development Goals and general principles laid down in the National and State policies, including the National Health Policy, 2002, and National Population Policy, 2000.
The ‘architectural correction’ of the health sector is a key objective for the NRHM, to be carried out through integration of vertical programs and structures; delegation and decentralization of authority; involvement of Panchayati Raj Institutions and other supportive policy reform measures in the areas of medical education, public health management, incorporation of Indian Systems of Medicine, regulation of healthcare providers, and new health financing mechanisms.
**Objectives(8)**
Reduction in child and maternal mortality Universal access to public services for food and nutrition, sanitation and hygiene, and elimination by universal access to public healthcare services, with emphasis on services addressing women's and children's health and universal immunization Prevention and control of communicable and non-communicable diseases including locally endemic diseases Access to integrated comprehensive primary health care Population stabilization, gender and demographic balance Revitalize local health traditions and mainstream AYUSH Promotion of healthy life styles
**Strategies**
A. Core
Decentralized village and district level health planning and management Appointment of Accredited Social Health Activist (ASHA) to facilitate access to health services Strengthening the public health service delivery infrastructure Mainstreaming of AYUSH Improved management capacity to organize health systems and services in public health Promoting non-profit sector to increase social participation and community empowerment, healthy behavior and inter-sectoral convergence
B. Supplementary
Regulation of the private sector, to improve equity and reduce out-of-pocket expenses Foster public private partnerships to meet national public health goals Reorientation of medical education Introduction of effective risk pooling mechanisms and social insurance to raise the health security of the poor, taking full advantage of local health traditions

The NRHM framework represents a conscious decision to strengthen public health systems and the role of the state as a healthcare provider [Figure 1]. It also recognizes the need to make optimal use of the non-governmental sector to strengthen public health systems and has increased access to medical care for the poor. In order to meet the core objectives of NRHM [Table 1], increasing the public expenditure on healthcare from 0.9% of GDP to 2 – 3% of GDP in an effective manner, through an increase in the central government budgetary outlay on health and ensuring a matching increase of the states' expenditure on health by at least 10% annually is one of the major steps.(3) As a corollary, the state health sector is required to develop capacities to absorb this additional fund flow.

**Table 1:** Statistical targets of NRHM

Targets	2012 A.D.
Infant mortality rate	30 / 1000 live births
Maternal mortality ratio	100 / 10000 live births
Total fertility rate	2.1
Malaria mortality reduction	50% by 2010, additional 10% by 2012
Kala Azar mortality reduction	100% by 2010, sustain elimination till 2012
Filaria/Microfilaria reduction	70% by 2010, 80% by 2012, elimination by 2015
Dengue mortality reduction	50% by 2010 and sustaining that level till 2012
Leprosy prevalence	< 1/10,000
Tuberculosis	Maintain 85% cure rate
Utilization of FRUs in terms of bed occupancy	> 75%
AS HA	4,00,000

**Figure 1:**
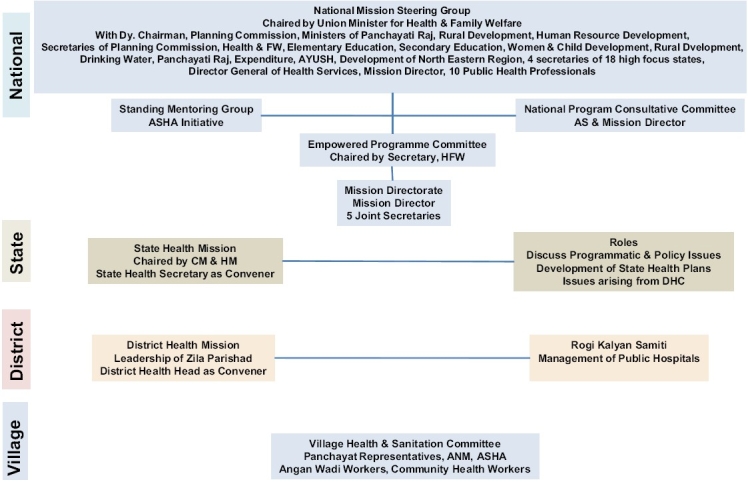
Organizational structure of the National Rural Health Mission

The core objective of NRHM is to create fully functional health facilities within the public health system. It is expected to provide a certain service guarantee at each level of the healthcare delivery system starting from a Subcenter (SC) to a District Hospital (DH).(3) The structural correction of the health system under NRHM is based on the following five principles. (1) Setting norms and standards and achieving service guarantees, (2) Innovations in the human resource development for the health sector, (3) Increasing participation and ownership by the community, (4) Improving the management capacity and (5) Flexible financing.

In a large country with inter-regional and intra-regional variations and inequalities, it is a challenging task to review the overall scenario of the mission's status in the country. However, the performance of NRHM can be reviewed under the following ten components.

## 1. Infrastructure and Preparedness of Health Facilities

Revamping of health infrastructure has been one of the important aspects of NRHM. The Sub-Health Centers (SHCs), Primary Health Centers (PHCs), and Community Health Centers (CHCs) are being housed in own buildings, and due emphasis is being given to clean and green surroundings of the health centers. However, the growth has not been uniform. In states like Bihar, DHs and CHCs are given priority over PHCs and SHCs. Chhatisgarh, Jharkhand, and Orissa are yet to show any significant growth in infrastructure. Promising work is being done in Assam, Bihar, Madhya Pradesh, and Rajasthan, but the leading states in this regard are Tamil Nadu and Maharashtra. In some states, the location of SHCs and PHCs being a little far from the village in deserted places, have been a cause of underutilization. In the country as per the Bulletin of Rural Health Statistics 2007, the total number of functioning SHCs, PHCs, and CHCs are 145272, 22370, and 4045, respectively.(4)

Twenty-four hour functional (24 × 7) health facilities are being provided at 12166 units (include SHCs, PHCs, CHCs), but 58% of these are in the non high focus large and small states. In the last three years (2005-2008) the number of PHCs with 24 × 7 facilities has increased from 1263 to 6397 and the highest growth has been in the larger states; both high focus and non high focus; the North Eastern (NE) states and non high focus states are yet to pick up. Similarly 24 × 7 functioning CHCs have increased from 980 to 2469 and the high focus non NE states have recorded 500% growth in this number, in the same time period. In a total of 623 districts in the country, 491 district level, 602 subdistrict level, and 1170 CHC level First Referral Units (FRUs) are functional, however, only 475 district hospitals are functioning as FRUs.(5)

Mobile medical units (MMUs) are working in just 243 of the 623 districts. Jharkhand and Madhya Pradesh are the two states among the high focus non-NE states that have provided mobile medical units in 24 and 20 districts, respectively. Under NRHM, all the NE states have operationalized MMUs. Most of the non focus states, except Maharashtra, had MMUs since the pre-NRHM days.

## 2. Case Load Handling by the Health System

There is an overall quantitative improvement in case load handling. It is evident from the data that the Outpatient Department (OPD) attendance has increased from 342 million in 2006-2007 to 477 million in 2007-2008. Similarly, the indoor patient load has gone up by 155% in the same time period, from 15 million to 23 million. The high focus non-NE states have shown 147% increase, and the other large states have shown a 170% rise. It is yet to pick up in the NE states. Jammu and Kashmir and Madhya Pradesh have shown three times the increase in OPD attendance at district hospitals. Similar improvement is evidenced in OPD attendance at the PHCs of these two states. Performance has been below par in Orissa and Himachal Pradesh. Data is not available from Rajasthan and Uttar Pradesh. Among the NE states, Manipur and Tripura have shown promising improvement. It is important to note that high priority states have poorer infrastructure and much less OPD attendance to begin with, thus, after the mission, the improvement of the facilities and the scope and proportion for increase in OPD attendance could have been much more, but the results do not reflect such changes. While in large non high focus states, where the status of utilization of services is already better, the resultant proportion of increase in OPD attendance is higher. This indicates a mismatch in efficient application and utilization of the mission funds in the states. However, the same is not true for the non high focus states, except for Kerala. Nevertheless, for a majority of the non high focus states, data is not available for 2006-2007. Wherever there has been posting of doctors and other staff and drugs have been made available, the utilization has gone up considerably.

The disadvantage has been in the form of overburdening of the health facilities. With increased institutional delivery, the case load has risen considerably. It has increased from 108 lakhs in 2005-2006 to 143 lakhs in 2007-2008. It is also worth noting that the increase in institutional deliveries in the high focus non-NE states has been 164% and about 200% in the NE states, in the same time period. The rise has been marginal in large states, from 64 to 72 lakhs, and actually a fall from 2.7 to 2.25 lakhs in small states and union territories (UTs).(5)

Besides overburdening the facilities, the other threat it poses is to the quality of service that is being provided to the consumers. At no place it is possible to keep the mother and baby for 48 hours after delivery. The reasons being, less number of beds, lack of food, and lack of shelter for escorts. In fact, most women prefer to go back home after receiving the payment. Janani Suraksha Yojana (JSY) beneficiaries have increased from seven lakhs in 2005-2006 to 72 lakhs in 2007-2008. The increase in high focus NE states has been from 1.74 to 45.24 lakhs.(5) Other states have also shown a similar growth.

## 3. Quality of Services

Quantitative improvement in services having been achieved in a majority of the states, the quality needs scrutiny. In fact a disproportionate increase in quantity without a proportionate increase in manpower and physical facility has led to a compromise in quality. The time spent per patient is limited, and emphasis on interpersonal communication and utilization of the health facility visit for health awareness has been negligible. On one side where JSY has brought pregnant women to institutions for delivery, the recommended 48 hour stay in most places could not be ensured. At some hospitals and health centers episiotomy is performed by AYUSH doctors who are not trained in surgical skills. If the quality of service decreases and the quantum of patients cannot be managed, then it may have a negative impact on the system. The brand NRHM that has succeeded in winning over the trust of clients who have been attracted to the health centers may end up in disappointment and carry a negative message back to the society. Incompetence in the early stage may lead to major aversion, which may be even more difficult to overcome for a long time.

## 4. Diagnostics and Drugs

In the NRHM, it is envisaged to provide basic laboratory testing facility at the PHC level and more advanced services at the CHC and DH levels. Surprisingly, the NRHM state data sheets do not contain information about laboratory facilities and its utilization. Basic drugs have been made available up to the SHC and PHC level in most places. However, AYUSH doctors are not provided with AYUSH medicines, and they are still prescribing modern medicinal drugs.

## 5. Human Resource Planning

There is acute shortage of all categories of staff in health sectors across the length and breadth of the nation. Most glaring are the lack of specialist doctors, laboratory technicians, and male health workers. A need for a second Auxiliary Nurse Midwife (ANM) is felt in all the states. According to the Bulletin of Rural Health Service on 31 December, 2008, 14851 SHCs had no ANM, 130812 had one ANM, and 25743 had two ANMs. At CHCs, 5117 specialists were posted against a requirement of 16180. The gap was highest in the high focus NE states where only nine positions were filled against a demand of 868.(4)

Among the doctors posted at PHCs, some are clinical specialists. They are unable to practice their clinical specialty and do not have the desire and aptitude for public health related work, as well as, lack managerial and administrative skills. They just wait for a transfer to a district hospital or a place of their choice. Such discrepancies on one side contribute to wastage of skilled manpower and on the other side leave a lot of public health activities unimplemented. Multi-skilling and multitasking is being talked about, but could be seen only among a handful of self-motivated doctors.

The Accredited Social Health Activist (ASHA) became the flag bearer of NRHM in rural India. As on 31 December, 2008, 500532 ASHAs have been selected. Of these 407957 were in the high focus non-NE states and 48552 in the high focus NE states. The goal of appointing ASHAs has been achieved to a large extent. Training of ASHA up to the third module has been completed, except in Bihar.(6) In villages of all the EAG states ASHAs are active and have been instrumental in improving institutional deliveries. This has streamlined the implementation of JSY in the EAG states. However, the strategic shift in institutional deliveries and reliance on ASHAs has led to resentment among the TBAs. On the other hand the ASHAs have started demanding full-fledged employment. There are documented evidences that ASHAs have encouraged institutional deliveries and are willing to take more work load. However, on the flip side, ASHAs have also concentrated their focus on institutionalization of delivery because of the monetary gains, thus ignoring the others tasks expected of them. The ASHA quarterly newsletter, ASHA sammelan, and annual ASHA awards are the highlights of the ASHA-related activities in Uttar Pradesh.(6) The ASHA support system and ASHA mentoring group is being formulated. Overburdening of ASHAs with additional work is another problem. At present 16 specific tasks are assigned to them, which are considered to be beyond the capacity of an ASHA. In some places resentment is brewing among ASHAs due to the delay in payments.

## 6. Financial Management

There are two new aspects in financial management. (1) Provision of flexi funds and freedom of the local governing body to spend the money according to local needs. (2) The creation of a mechanism for the rapid transfer of funds to peripheral institutions. Some of the states have been able to create the system of electronic transfer of funds with the help of banks. In these states utilization has also begun. Although in other states, transfer of funds is slow, irregular, and erratic. This is due to the noncreation of Rogi Kalyan Samitis (RKS) and Village Health and Sanitation Committees (VHSC); thus funds have remained idle or unutilized. Another hurdle is lack of focus and understanding of administrative issues by the Medical Officers at the PHC level.(6)

The number of joint accounts operational at SHC and VHSC in high focus non-NE states is 136901. Bihar is the only state where no joint accounts are opened.(6) Another glaring deficit is the inability of the states to utilize the available funds. By now the central government has released Rs 28408 crores up to 2008-2009, of which Rs 11205 crores remains unused.

## 7. Community Processes Including Community Participation

NRHM has emphasized on community participation in a big way. It is expected that community leaders will participate in the governance and improvement of the health facilities of the area. The untied funds will be utilized with the consensus of the community; but the process of community participation is slow.

Rogi kalyan samitis have been formed as follows: DH-565, CHCs-3912, PHC-16628, others-1995. In large states like Uttar Pradesh the participation of the Panchayati Raj Institutions (PRIs) is not very encouraging. Lack of political will and an attitude of indifference prevails. Meetings of VHSC are not held regularly and the RKS are also not very proactive in participating in the activities of the health institutions.(6) In some places the process of utilization of untied funds is yet to begin. The Medical Officer in-charge, because of his clinical orientation, is busy with patient care, does not have the foresight to develop the center or provide public health services.

For the PRIs, local health institutions are not a priority. Development projects and funds disbursement activities get priority over health concerns. Lack of awareness and motivation has led to minimal community participation. It appears to be an uphill task to initiate a social audit at the village level.

## 8. Difficult Areas and Vulnerable Groups

Difficult areas are hard to reach areas, located in difficult terrains. Vulnerable groups are the marginalized population namely tribals and schedule castes and families living below poverty levels. In some states insurgency has made it difficult for the health workers to function. Mobile medical units and ‘difficult area allowance’ are some of the steps initiated, for improving the services in difficult areas. Monetary incentives and faster promotions have been the other perks being offered to health workers in difficult areas. In Maharashtra, an additional stipend of Rs. 1000 per month is given for working in tribal areas, and an additional payment of Rs. 1500 is given to ANMs, for working in insurgency affected areas of the Nagpur division.(6)

## 9. Information Systems and Record Maintenance

Integrated Disease Surveillance Project (IDSP) and Health Management Information System (HMIS) were aimed at documentation and flow of data, for generation of meaningful indicators of process, and impact of the various components of public health in the umbrella of NRHM. On the one hand HMIS was expected to establish a data management system that would improve governance, as well as, a monitoring system. However, the report of the second Common Review Mission pointed out that the analysis and use of information was very weak. Computers have been made available at least up to the block level in all states and even up to the level of PHCs in Kerala, Maharashtra, and Tamil Nadu.(6) Although in various places data entry operators are not appointed or training of manpower has not yet been done. In some places severe shortage of electricity and lack of good internet connectivity have been common hurdles in the successful implementation of HMIS as well as IDSP. It is the general outcome in most of the states that management of available data and analysis of the same is very poor and the validity and reliability of the data is questionable. The two common reasons for this wide gap in HMIS are that the indicators are used rather than the data. The system is not geared to analyze and display data at a peripheral level. The other reasons are frequent changes in recording and multiplicity in data reporting. Vertical programs have their own formats of recording and reporting particularly in the Revised National Tuberculosis Control Program (RNTCP), National AIDS Control Program (NACP), and National Vector Borne Diseases Control Program (NVBDCP). Tamil Nadu and Maharashtra have good computerized HMIS systems, but most of the states still rely on manual preparation of reports, hence there is delay in transmission and hardly any analysis is undertaken. Assam, Jharkhand, Kerala, and Mizoram have been found to have very weak HMIS.(6)

## 10. Innovations in Implementation

A wide range of innovative approaches are being implemented by states to address identified needs/specific gaps in health services. Two hundred and eighteen such innovations are documented by the Mission for its stakeholders and for cross learning purposes.(7) Innovations have been made in the field of the following nine major themes. Some of the promising innovations are listed below.

### 10.1. Safe motherhood

Chirnajeevi Yojana in Gujarat is a health financing scheme covered through a public–private partnership for emergency obstetric care and emergency transport services for women belonging to the below poverty line (BPL) category. In this scheme, 852 out of 2000 obstetricians/gynecologists are already enrolled. *Ayushmati Scheme* in West Bengal, *Mamta Friendly Hospital Initiative* in Delhi, and *Saubhagyawati Scheme* in Uttar Pradesh are similar schemes.

Birth Waiting Rooms in Andhra Pradesh is a pilot intervention to ensure that pregnant women from distant tribal areas reach the institutions a couple of days before the expected date of delivery, in order to avoid complications. Initially three waiting rooms were created in each district. *Vande Mataram Scheme* in West Bengal is a scheme which involves the private sector in providing safe motherhood and family planning services. Enrolled *Vande Mataram* physicians are provided a kit of Iron and Folic Acid tablets, condoms, Oral Contraceptive Pills, and Intra Uterine Devices for free distribution. For transportation of obstetric emergencies, *Janani Express Yojana* in Madhya Pradesh, free bus passes in Andhra Pradesh, *Janani Suraksha Vahini* in Karnataka, and various ambulance services are launched in several states.

### 10.2. Immunization and infant and young child feeding

*Ankur Project* in Maharashtra provides home-based new born care by a community health worker based on the *Gudchiroli* model. *Kano parbo na* in West Bengal uses a positive deviance approach to identify feeding practices of mothers with healthy children and transmit them to other mothers through a community-based approach, for reduction of low birth weight and malnutrition. In Rajasthan a *Panchamrit campaign* – a week long intervention program during the first three months of the year covers five services, including immunization. *Balshakti Yojana* in Madhya Pradesh identifies grade III and IV malnourished children by ICDS functionaries, who are treated in nutritional rehabilitation centers.

### 10.3. Adolescent reproductive and sexual health

*Saathiya Youth Friendly Project* in Uttar Pradesh strengthens provider knowledge and skills on contraception and sexual health for out-of-school girls at youth information centers.

### 10.4. Behavior change communication

*Goli ki hamjoli* campaign in seven states helps in increasing the use of low-dose Oral Contraceptives among young married middle class women in 33 towns of north India. *Bindaas Bol* is a campaign directed at promotion of condom use by men, in nine states. The campaign that was funded by USAID and implemented by ICICI bank, used an integrated communication and marketing approach involving mass media campaigns, celebrity involvement, innovations to reach retailers, partnership with condom manufacturers, marketers, and health providers. *Saathi Bachpan ke* promotes the use of Oral Rehydration Salt for diarrhea in 33 towns of eight states of North India.

*Aadarsh Dampati Samman* in Uttar Pradesh awards cash of Rs. 500 to two men per subcenter every year for following the two child norm and adopting the male sterilization method, to showcase them as role models, to induce a behavior change with regard to male participation in family planning. *ASHA radio* in Assam distributed radios, and programs for ASHA are broadcasted on All India Radio, twice a week, providing health education information to the ASHAs. Pre-paid postcards have also been distributed to the ASHAs for giving a feedback.

*Bodhana Nauka / Information boat* is a Kerala campaign, intended for people living in the water logged Kuttanad region of Alappuzah district for creating awareness about water borne and vector borne diseases.

### 10.5. Gender mainstreaming

Gender Budgeting in Karnataka has made special provisions in the budget for needs of adolescent girls, pregnant, and lactating women in tribal districts. The *Bhagya Lakshmi* scheme in Karnataka and *Ladli* scheme in Delhi gift Rs. 10000 in the form of fixed deposit, which can be encashed after attaining 18 years of age, to a girl child born in a BPL family.

### 10.6. Service delivery for RCH

Mobile Health Clinics in seven states, mobile boat clinics in Assam, Mobile Helicopter services for remote inaccessible areas in Tripura, and floating dispensaries in the Narmada basin of Maharashtra are some of the services rendered to the difficult-to-reach / inaccessible areas. Social franchising networks are functioning for distribution of contraceptives in Karnataka, Uttar Pradesh, Jharkhand, and Uttarakhand. Several insurance schemes are operational for BPL families to meet the cost of their illnesses in Goa, Madhya Pradesh, Karnataka, Uttarakhand, Jharkhand, Andhra Pradesh, and Mizoram.

### 10.7. Program management

Cash incentives for workers are launched in various states, for different categories, for working in remote areas, disturbed areas, and with tribal populations. In Uttarakhand, mobile phones are provided to ANMs in selected districts. In Assam, performance-based incentives are given to the team conducting institutional deliveries. In Maharashtra, a cash incentive is given to panchayats for improving the status of grade III and IV malnourished children. Innovative program management initiatives include decentralized clinical training and post training supervision of Integrated Management of Neonatal and Childhood Illnesses (IMNCI) trained personnel by Non-Governmental Organizations (NGOs); community and *panchayat* involvement in the planning, monitoring, and management of health services and facilities and program monitoring and management information systems. There have been improvements of procurement and finance systems, for example, Tamil Nadu and Kerala Medical Services Corporation as a drug management and supply system, e-banking for fund management in Kerala and Debit cards for ASHAs.

A summary of the status of and key facts and figures related to NRHM as on 31 December, 2008 is given in Table 2.

**Table 2:** Status of NRHM: Key facts and figures as on 31 December, 2008

Parameters	Total	High focus non-NE states	High focus NE states	Non-high focus large states	Non-high focus small states and UTs
Number of districts	623	298	87	217	21
District Hospitals	570	292	72	183	21
Rogi Kalyan Samitis registered					
District Hospitals	565	290	74	187	14
Community Health Centers	3912	1801	212	1885	14
PHCs	16628	5593	1404	956	65
Others	1995	762	55	1176	2
ASHAs recruited		407957	48552	41516	2507
24 × 7 functional health facilities	12166	4367	739	6959	101
24 × 7 functional PHCs	7212	2665	525	3971	51
24 × 7 functional CHCs	2690	1004	192	1479	15
District level FRUs	491	238	56	182	15
AYUSH facilities	7275	2626	339	4257	53
District Project Management Units	576	289	87	182	18
Institutional deliveries (lakhs)					
2005 – '06	108.41	38.68	2.51	64.55	2.67
2007 – '08	143.16	63.81	4.42	72.68	2.25
JSY beneficiaries (lakhs)					
2005 – '06	7.04	1.74	0.27	5.00	0.04
2007 – '08	72.01	45.24	3.71	22.93	0.13
Districts with Mobile Medical Units	243	50	70	112	11
OPD case load (lakhs)					
2006 – '07	3424.67	486.19	13.13	2851.04	73.32
2007 – '08	4779.93	817.58	23.21	3894.52	44.60
IPD case load (lakhs)					
2006 - '07	154.7	53.78	1.5	97.12	2.16
2007 - '08	239.96	83.32	4.0	151.59	1.07
Leprosy prevalence rate /10 000	0.78	0.97	0.33	0.71	0.81
Deaths due to malaria (2008)	768	193	296	279	0
Deaths due to kala azar	137	133	1	3	0
Deaths due to suspected JE	644	563	78	3	0
Deaths due to dengue	80	6	0	72	2
Chikunguniya cases	2262	21	0	2228	13
TB case detection rate (%)	72	62.6	NA	64.9	66.14
TB cure rate (%)	87	87.7	NA	85.3	90.29
District IDSP units functional	399	151	40	194	14
IDSP trained personnel	1871	575	278	997	21

High focus non-NE states comprise of Bihar, Chhatisgarh, Himachal Pradesh, Jammu & Kashmir, Jharkhand, Madhya Pradesh, Orissa, Rajasthan, Uttar Pradesh, and Uttarakhand. The high focus North eastern states are Arunachal Pradesh, Assam, Manipur, Meghalaya, Mizoram, Nagaland, Sikkim, and Tripura. Small states and UTs are designated as non-high focus small and UTs namely Andaman & Nicobar, Chandigarh, Dadra & Nagar Haveli, Daman & Diu, Delhi, Lakshadweep, and Pudducherry. Rest of the states is grouped as non-high focus large states.

## Conclusions

National Rural Health Mission, in its endeavor to improve the healthcare delivery system in rural India may be considered as a paradigm shift in the way healthcare delivery is to be executed. Because of its mission mode, the execution moved at a fast pace and the professional, systematic approach adopted in the mission has shown its impact, albeit delayed and diverse. The visible impact has been in the form of revamping the infrastructure. Almost all states have taken up the task of quantitative and qualitative improvement in the healthcare delivery infrastructure from the grass root, from the Sub Health Center level to the District Hospital. The availability of funds and its hassle-free utilization by local governing bodies has also helped in bringing about desirable changes in the system. The single most visible impact has been the *Janani Suraksha Yojana*, in terms of the increase in the number of institutional deliveries across the country. ASHA the voluntary worker has certainly kindled hopes of a meaningful change in the Reproductive and Child Health (RCH) program through their very presence and enthusiasm for work. State level innovations in the RCH program, the funding mechanisms, and improved governance appear to be very promising. Mobile health clinics, transportation of pregnant women, and thrust on family welfare activities are the optimistic highlights of the program. However, the feel good features of NRHM end here. Accomplishing the goals of the mission have still a long way to go, and more innovations are required to meet the challenges, which several states are facing in making it a success.

It is a matter of concern that states that have been lagging behind in the pre-NRHM period have not shown remarkable changes. These states are still struggling to implement the NRHM in its letter and spirit. One of the biggest challenges is to meet the human resource requirement for the services to be delivered. There is a deficit of staff across the board, specialist doctors, male multipurpose workers, and laboratory technicians. A lot more work is to be done in order to improve the quality of healthcare, through multi-skilling and multitasking, by the care providers – the area that lags behind most significantly is the health management information system and the IDSP program. In spite of the supply of computers and availability of internet links, data management and information flow to and from the peripheral levels is still very poor. However, the future of the mission appears promising as a political will, hard work, and professional managerial approach will help cross the hurdles and accomplish the mission.
